# Handling drop-outs which imply a change of treatment: an evaluation of reference-based imputation

**DOI:** 10.1186/1745-6215-16-S2-P153

**Published:** 2015-11-16

**Authors:** Ian White, Royes Joseph, Nicky Best

**Affiliations:** 1 MRC Biostatistics Unit, Cambridge, UK; 2 GlaxoSmithKline, Uxbridge, UK

## 

In some randomised trials, loss to follow-up is associated with end of treatment. This applies particularly, but not exclusively, to pharmaceutical trials. In this setting Carpenter *et al* (Journal of Biopharmaceutical Statistics 2013; 23: 1352-71) distinguished the “de facto” estimand, the mean of the observed and unobserved outcomes in the actual treatment circumstances of the trial, from the “de jure” estimand, the mean of the observed and unobserved outcomes if treatment were maintained after loss to follow-up. Standard missing at random analysis estimates the de jure estimand. Carpenter *et al* (2013) proposed “reference-based imputation” methods to estimate the de facto estimand. These involve various ways to use data from a reference arm (usually the control arm) to multiply impute post-dropout outcomes. However, the properties of these methods have been little explored.

This talk starts by reviewing the reference-based imputation methods. We then use a potential outcomes framework to identify the methods’ implicit assumptions about the causal effect of treatment. Firstly assuming that the covariance structures are the same across arms, we show that reference-based imputation methods are equivalent to specific assumptions about how the treatment effect decays after end of treatment: “jump to reference”, “copy reference” and “copy increments in reference” respectively assume the treatment effect is eliminated, decays according to the covariance structure, and is maintained. We then show that bias is introduced if the covariance structures differ across arms. These results are illustrated in a simulation study and using a pharmaceutical trial.

